# Dose-response of different dietary leucine levels on growth performance and amino acid metabolism in piglets differing for aminoadipate-semialdehyde synthase genotypes

**DOI:** 10.1038/s41598-019-55006-z

**Published:** 2019-12-06

**Authors:** Micol Bertocchi, Paolo Bosi, Diana Luise, Vincenzo Motta, Chiara Salvarani, Anisa Ribani, Samuele Bovo, Aude Simongiovanni, Keiko Matsunaga, Tetsuya Takimoto, Makoto Bannai, Etienne Corrent, Luca Fontanesi, Tristan Chalvon-Demersay, Paolo Trevisi

**Affiliations:** 1 Department of Agricultural, Environmental and Food Sciences, University of Molise, Campobasso, Italy; 2 Department of Agricultural and Food Sciences, University of Bologna, 40127 Bologna, Italy; 3 Ajinomoto Eurolysine S.A.S., 75017 Paris, France; 4 Ajinomoto Co., Inc., Tokyo, 104-8315 Japan

**Keywords:** Metabolism, Physiology

## Abstract

Dose-response studies of dietary leucine (Leu) in weaners are needed for a proper diet formulation. Dietary Leu effect was assessed in a 3-weeks dose-response trial with a 2 (genotype) x 5 (diets) factorial arrangement on one-hundred weaned pigs (9 to 20 kg body weight (BW)). Pigs differed for a polymorphism at the aminoadipate-semialdehyde synthase (AASS) gene, involved in lysine (Lys) metabolism. Pigs received experimental diets (d7 to d28) differing for the standardized ileal digestible (SID) Leu:Lys: 70%, 85%, 100%, 115%, 130%. Daily feed intake (ADFI), daily gain (ADG) and feed:gain (F:G) in all pigs and ADG and F:G in two classes of BW were analyzed using regression analysis with curvilinear-plateau (CLP) and linear quadratic function (LQ) models. Amino acid (AA) concentrations in plasma, liver, muscle and urine were determined. AASS genotype did not affect the parameters. Dietary Leu affected performance parameters, with a maximum response for ADG and F:G between 100.5% and 110.7% SID Leu:Lys, higher than the usually recommended one, and between 110.5% and 115.4% and between 94.9% and 110.2% SID Leu:Lys for ADG for light and heavy pigs respectively. AA variations in tissues highlighted Leu role in protein synthesis and its influence on the other branched chain AAs.

## Introduction

The reduction of crude protein (CP) content in pig diets can be beneficial to reduce nitrogen environmental losses from the animals, when the dietary formulation is based on the use of the net energy system and balanced AA^1^. This implies a consequent supplementation with synthetized essential amino acids (AA) to maintain performance^2^. Thus, AA requirement estimation has become pivotal in diet formulation to allow for a high feed efficiency and to maximize feed intake and growth rate in growing pigs, which implies the importance of knowing the metabolism of each AA and role also considering different pig genotypes and phenotypes^3^.

Leucine (Leu) is a branched-chain amino acid (BCAA) that plays a role not only as substrate for protein synthesis but also as “functional” AA. By acting as a signal molecule in the first step of mTOR signaling pathway, Leu stimulates protein deposition in skeletal muscle^4–6^. Leucine is also involved in tissue absorption of other AA such as lysine (Lys), which passes the cell membrane through b^0,+^ (main cationic AA transporter exchanging Leu with Lys in small intestine). The b^0,+^ gene expression seems to be decreased in the gut by dietary Leu excess^7,8^. Furthermore, Leu plays a central role in the catabolism of the three branched-chain amino acids (BCAAs) (namely, Leu, Valine (Val) and Isoleucine (Ile)). BCAAs share common isoenzyme complexes in the first two steps of their catabolism. The first one is a reversible transamination that produce branched-chain α-keto-acids. These products enter in the second step for an irreversible decarboxylation, which is rate limiting for BCAA catabolism. In this second step the key-enzyme complex branched-chain α-keto acid dehydrogenase (BCKDH) is regulated by the dietary Leu supply. Thus, it was observed that an increase in BCKDH activity (*i.e*. due to Leu excess) increases the catabolism of the three BCAAs, even in case of Val and Ile deficiencies^9^. As consequence, a diet with an excess of Leu affected availability of BCAAs for protein synthesis and impaired feed intake^8–12^. Leu level seems to be correlated to the decrease of feed intake directly through its stimulation of mTOR signaling pathway in hypothalamus^13^ and indirectly through the activation of GCN2 signaling pathway in response to the lower availability of the other BCAAs^14,15^.

Since Lys is the first limiting AA in pigs, pig diets are formulated to meet Lys recommendations and they may contain excess of Leu, especially in corn-based diets^8^. Moreover, in the today European context of reducing the dietary CP level for environmental and animal health reasons, Leu may represent as the next limiting AA after Lys, Threonine (Thr), Methionine + Cysteine (Met + Cys), Tryptophan (Trp) and Val, especially in wheat and barley-based diets. Thus, it is important to consider the ratio of Leu to Lys. Furthermore, Leu requirement in piglets is not well defined due to limited data: National Research Council (NRC)^16^ defined a standardized ileal digestible (SID) Leu to Lys requirement of 100%, but recent studies reported different results about Leu requirements in growing pigs. Estimates of SID Leu:Lys requirement of 102% and 108%, were proposed by Gloaguen *et al*.^17^ and Wessels *et al*.^18^ in pigs weighting 10–20 kg, and a range between 100 and 120% by García *et al*.^8^ in pigs of 28–42 kg of BW, while Soumeh *et al*.^19^ determined a minimum of 93% SID Leu:Lys to increase pig growth in pigs of 9–15 kg of BW. However, the effect of age or live weight was not specifically considered in the previous studies.

Considering the potential consequences of dietary Leu excess and the need to estimate Leu requirement in piglets, data on the response of piglets to Leu must be collected. Dose-response experiments are commonly used to define the minimal dose which corresponds to specific responses; this can be used to decide the practical recommendation of the nutrient^20^. In these studies, statistical nonlinear models are commonly used, a.k.a. linear-plateau (LP) and curvilinear-plateau (CLP) models. Nevertheless, the linear quadratic function (LQ) should also be taken into account as an alternative as this model considers the possibility of a declining growth when exceeded the required dose of the tested nutrient^18^. This effect is not captured by nonlinear models.

The important role of Leu as essential AA but also as “functional” AA influencing Lys absorption^7,8^ implies that Leu requirement may interact with the one of Lys. Lysine efficiency for growth is partially limited due to its irreversible catabolism, accounting for about 10–14% of the available dietary Lys in growing pigs^21^. Most of the catabolic process occurs in the liver through the saccharopine α-aminoadipate d-semialdehyde pathway. However, a great capacity for Lys degradation through the same pathway has been also discovered in non-hepatic tissues, such as intestine and muscle^22,23^. The two key enzymes of the pathway, lysine α-ketoglutarate reductase (LKR) and saccharopine dehydrogenase (SDH), are catalyzed by the bifunctional protein aminoadipate-semialdehyde synthase *(AASS)*^24^.A genome wide association study conducted by Fontanesi *et al*.^25^ on a large sample of Large White pigs revealed a quantitative trait locus (QTL) for Lys concentration in blood in adult animals in a chromosome region including the *AASS* gene.

Basal oxidation of the absorbed dietary Lys is one of the main accountable for Lys inefficiency in protein deposition. Moreover, this degradation mainly depends on the bifunctional protein *AASS*. Therefore, modification or inhibition of the functions of this enzyme could reduce Lys degradation, directing this AA towards protein synthesis and reducing its requirement^23,24^.

## Aim

The main aim of this study was to evaluate the effect of different dietary levels of SID Leu with a fixed SID Lys supply (i.e. SID Leu:Lys) on piglet growth performance and AA metabolism to assess Leu requirement in the weaned pig. Moreover, we also wanted to investigate if the SID Leu:Lys requirement changes in weaned pigs with different QTL genotypes on the *AASS* gene.

## Results

All the piglets remained healthy without signs of diarrhea during the whole experimental period, except that one piglet (100% SID Leu:Lys group) did not grow and was excluded from the trial.

The effect of the different Leu levels on growth performances of the piglets is shown in Table 1. Average daily gain (ADG) was significantly affected by the diets for every week after the diet differentiation (d7) (*P* *=* *0.0*22 for week 2 and *P* *<* *0.0001* for the others) and for the whole experimental period (d7 - d28), increasing from 70 to 115% SID Leu:Lys group (*P* *<* 0.0001). From week 3 (d14) until the end of the trial, 130% SID Leu:Lys group showed a decrease of ADG compared to the pigs fed 115% SID Leu:Lys diet(*P* *≤* 0.0001). Average daily feed intake (ADFI) was significantly affected by the diets from week 3 (d14) to the end of the trial and over the whole experimental period (d7 - d28) (*P* *<* 0.0001). ADFI was lower in the 70% SID Leu:Lys group than the others. The feed-to-gain ratio (F:G) was also affected by the diets for the whole experimental period (d7 - d28; *P* *<* 0.0001).

**Table 1:** Effect of the dietary SID Leu:Lys^1^ content and of the AASS^2^ gene region genotype on growth performances of the piglets.

Items	% SID Leu:Lys^1^							AASS^2^				% SID Leu:Lys^1^ × AASS^2^
	70	85	100*	115	130	SEM	*P*	AA	GG*	SEM	*P*	*P*
**Body weight (Kg)**												
d0^3^	8.30	8.38	8.49	8.29	8.41	0.33	0.992	8.56	8.18	0.21	0.194	0.938
d7	9.19	9.47	9.59	9.42	9.53	0.37	0.953	9.57	9.31	0.24	0.430	0.946
d28	16.22	19.64	20.95	21.04	20.36	0.81	0.0002	19.84	19.44	0.52	0.582	0.700
**Average daily gain (g/d)**												
week 1^3^	128	156	158	163	160	20	0.747	145	162	13	0.349	0.874
week 2	290^B^	409 ^A^	401 ^A^	412 ^A^	411 ^A^	30	0.022	392	378	20	0.620	0.429
week 3	314 ^C^	442^B^	536 ^A^	513^AB^	507^AB^	26	<0.0001	480	445	17	0.147	0.368
week 4	401 ^C^	601^B^	685^AB^	734 ^A^	629^AB^	35	<0.0001	596	624	22	0.357	0.457
d7-d28	335 ^A^	484^B^	541^B^	553^B^	516^B^	25	<0.0001	489	483	16	0.694	0.284
**Average daily feed intake (g/d)**												
week 1^3^	242	275	259	260	262	8	0.108	262	257	5	0.524	0.082
week 2	473	596	552	571	598	36	0.102	561	615	23	0.833	0.939
week 3	547^B^	772 ^A^	762 ^A^	766 ^A^	787 ^A^	40	0.0001	740	713	26	0.446	0.533
week 4	679^B^	979 ^A^	996 ^A^	1038 ^A^	988 ^A^	50	<0.0001	936	936	32	0.991	0.493
d7-d28	566^B^	782 ^A^	770 ^A^	792 ^A^	791 ^A^	39	0.0001	746	734	25	0.738	0.403
**Feed to gain**												
week 1^3^	1.89	1.76	1.64	1.60	2.95	1.16	0.323	1.81	1.59	1.38	0.563	0.475
week 2	1.63	1.46	1.38	1.39	1.45	0.46	0.200	1.43	1.63	0.30	0.398	0.883
week 3	1.74^B^	1.75^B^	1.42 ^A^	1.49 ^A^	1.55^AB^	0.12	0.002	1.54	1.60	0.08	0.158	0.172
week 4	1.69 ^C^	1.63 ^C^	1.45^AB^	1.41 ^A^	1.57^BC^	0.05	0.001	1.57	1.50	0.03	0.060	0.932
d7- d28	1.72^D^	1.62^CD^	1.42^AB^	1.44 ^A^	1.55^BC^	0.03	<0.0001	1.54	1.56	0.02	0.594	0.152

Interactions between diet and genotype not statistically significant.

^-^Means on the same line with different superscripts differ for *P* < 0.01.

^*^One piglet did not grow and was excluded from the trial. ^1^Standardized ileal digestible leucine to lysine ratio. ^2^The two homozygous genotypes for the aminoadipate-semialdehyde synthase (*AASS*) gene marker. ^3^The piglets received a common prestarter diet during the first week and then the diets differing by their SID Leu:Lys content from d7 to d28.

No statistically significant interaction between diet and *AASS* genotypes was found. The *AASS* genotype did not affect growth performance (Table 1).

**Figure 1:**
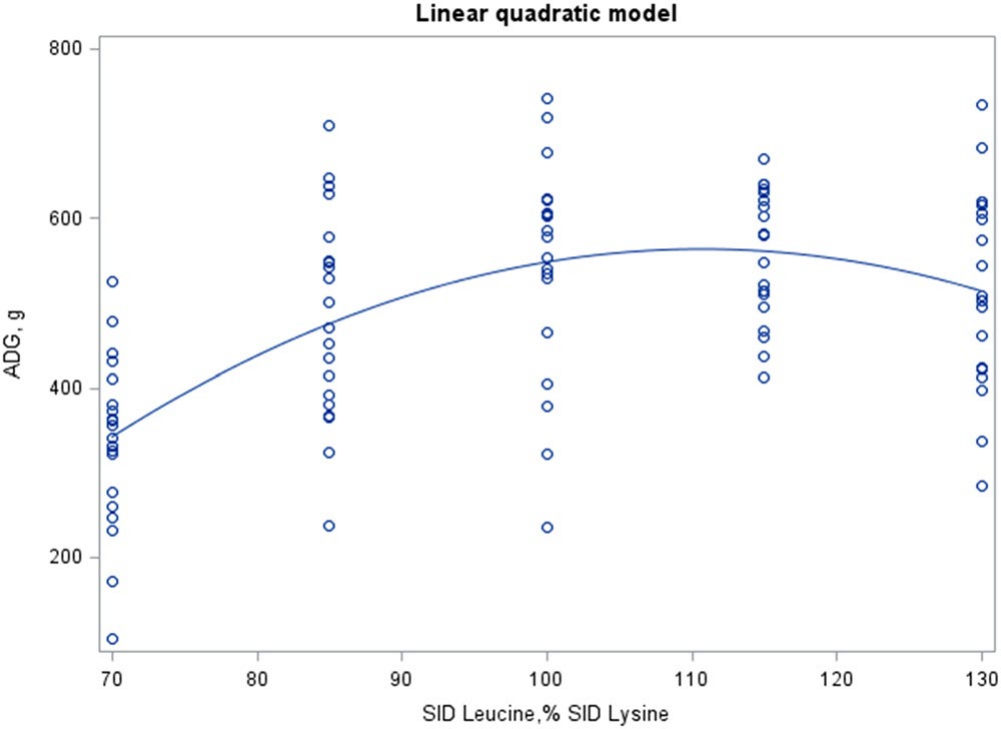
Estimation of leucine requirement for weaned piglets. Prediction of the average daily gain (ADG) according to the standardized ileal digestible (SID) leucine to lysine dietary content with a linear quadratic model. Observed values (o) from d7 to end (d28). Equation of prediction (parameters ± S.E.): Y = −1061 (±285) + 29.46 (±5.89)*X – 0.133 (±0.029)*X^2^. Adjusted R^2^ = 0.334; Root mean square error (RMSE) = 96. For all the parameters, P < 0.0001. Estimated maximum ADG at 570 g with 110.7% SID leucine to lysine.

Figure 1 shows the prediction of the ADG according to the dietary SID Leu:Lys content by LQ model, with a value of 110.7% SID Leu:Lys to maximize ADG (570 g/d). We tested also the ADG prediction by CLP model (Supplementary Fig. 1), which indicated a value of 100.5% SID Leu:Lys at the inflection point of maximum ADG (538 g/d).

**Figure 2:**
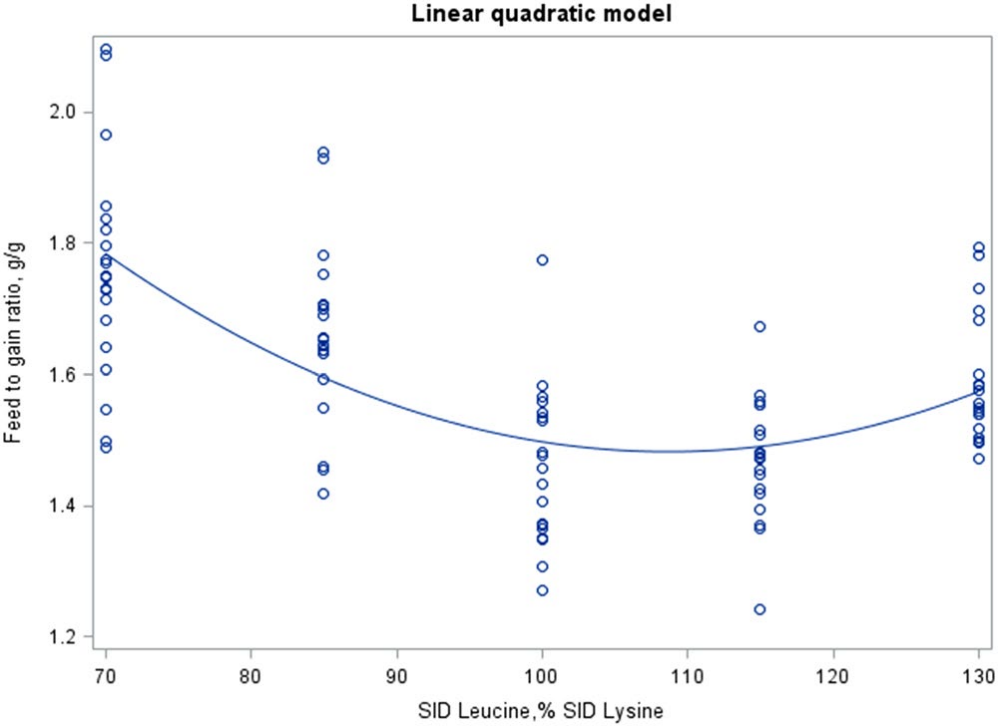
Estimation of leucine requirement for weaned piglets. Prediction of feed to gain ratio according to the standardized ileal digestible (SID) leucine to lysine dietary content with a linear quadratic model. Observed values (o) from d7 to end (d28). Equation of prediction (parameters ± S.E.): Y = 3.867 (±0.335) − 0.0439 (±0.0069)*X + 0.000202 (±0.000035)*X^2^. Adjusted R^2^ = 0.401; Root mean square error (RMSE) = 0.163. For all the parameters, P < 0.0001. Estimated minimum feed to gain ratio at 1.48 g/g with 108.7% SID leucine to lysine.

Figure 2 represents the prediction of F:G ratio according to the SID Leu:Lys dietary content by LQ model, which predicted a value of 108.7% SID Leu:Lys at the minimum point of F:G (1.48 g/g). We also tested the F:G prediction by CLP model (data not shown) but data did not properly fit because the predicted inflection point was over the range of the tested SID Leu:Lys levels.

The prediction curves for ADG and F:G from the SID Leu:Lys dietary content obtained for the two *AASS* genotypes, testing the intercept for each genotype, are overlapping (Supplementary Fig. 2).

**Figure 3:**
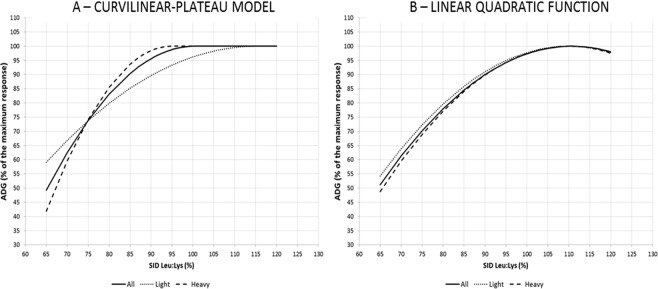
Estimation of leucine requirements in 2 different body weight classes: Light pigs (• • •), heavy pigs (− − −). Percentage of the maximum response of average daily gain (ADG) according to the standardized ileal digestible (SID) leucine to lysine dietary content predicted by curvilinear-plateau model (**A**) and linear quadratic function (**B**), from d7 to end (d28). (A) Equation of prediction in light pigs (• • •) before inflection (parameters ± S.E.): Y = −556 (±586) + 18.04 (±12.90)*X – 0.0782 (±0.0687)*X^2^. Adjusted R^2^ = 0.248; Root mean square error (RMSE) = 91; Estimated plateau = 485.0 g of ADG with 115.4% SID leucine to lysine. Equation of prediction in heavy pigs (− − −) before inflection (parameters ± S.E.): Y = −2877 (±1466) + 73.1 (±35.8)*X −0.385 (±0.216))*X^2^. Adjusted R^2^ = 0.496; Root mean square error (RMSE) = 63; Estimated plateau = 592.6 g of ADG with 94.9% SID leucine to lysine. Equation of prediction in all pigs (──) before inflection (parameters ± S.E.): Y = −1653 (±905) + 43.61 (±21.44)*X – 0.2170 (±0.1244)*X^2^. Predicted inflection point at 538 g ADG with 100.5% SID leucine to lysine. Adjusted R^2^ = 0.327; Root mean square error (RMSE) = 96. (B) Equation of prediction in light pigs (• • •) (parameters ± S.E.): Y = −844 (±377) + 24.2 (±7.8)*X − 0.110 (±0.039)*X^2^. Adjusted R^2^ = 0.276; Root mean square error (RMSE) = 88; Estimated maximum ADG at 495.6 g with 110.5% SID leucine to lysine. Equation of prediction in heavy pigs (− − −) (parameters ± S.E.): Y = −1280 (±342) + 34.57 (±7.01)*X – 0.157 (±0.035)*X^2^. Adjusted R^2^ = 0.463; Root mean square error (RMS)E = 67; Estimated maximum point at 625.4 g of ADG with 110.2% SID leucine to lysine. Equation of prediction in all pigs (──) (parameters ± S.E.): Y = −1061 (±285) + 29.46 (±5.89)*X – 0.133 (±0.029)*X^2^. Adjusted R^2^ = 0.334; Root mean square error (RMSE) = 97. For all the parameters, P < 0.0005. Estimated maximum ADG at 570 g with 110.7% SID leucine to lysine.

After the first preliminary week (week 1), Leu requirement for ADG was also estimated using two classes of body weight (BW), including all the pigs under and over the mean, respectively. Performance parameters for the two BW classes are reported in Table 2, where the BW classes significantly differ for ADG and feed intake (*P* *<* 0.0001) but not for F:G. Figure 3 reports the percentage of the maximum response for ADG according to the dietary SID Leu:Lys content using the LQ and CLP models. The LQ model estimated a Leu maximum response for light and heavy pigs at 110.5% and 110.2% SID Leu:Lys, respectively. Differently, the CLP model predicted a Leu maximum response at 115.4% and 94.9% SID Leu:Lys for light and heavy pigs respectively. Especially for heavy pigs, the maximizing dose of Leu diverged considerably from what was defined by LQ model. No effect of BW was seen in the intra-class analysis for the slopes and for the intercept. For F:G, the response for the two BW classes in the two models was similar (Supplementary Fig. 3).

**Table 2:** Growth performance in the two body weight (BW) classes of piglets.

Items and statistical significance	BW classes^1^	Mean	Standard error
BW at d7, Kg	heavy	10.5	0.19
*P* < 0.0001	light	7.9	0.20
ADG, from d7 to d28, g	heavy	534	14.1
*P* < 0.0001	light	432	14.7
Feed intake, from d7 to d28, g	heavy	822	20.7
*P* < 0.0001	light	651	21.7
Feed to gain, from d7 to d28, g/g	heavy	1.56	0.018
*P* = 0.256	light	1.54	0.019

^1^Two different BW classes (heavy and light pigs) composed of all pigs over (n = 50) and under the mean (n = 49), respectively.

**Figure 4:**
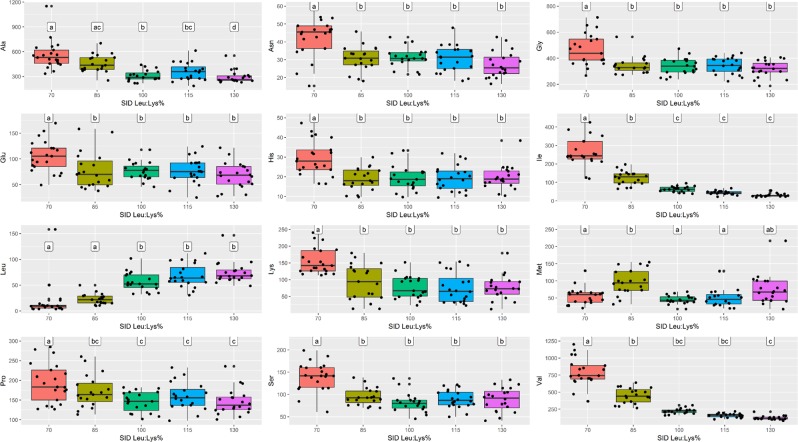
Amino acids concentration (μmol/l) in plasma at d21 associated to the different standardized ileal digestible leucine to lysine (SID Leu:Lys) dietary contents. Values represents the median, first and third quartile and 95% confidence interval of median of plasma AA of pigs fed diets with 70%, 85%, 100%, 115% and 130% SID Leu:Lys. Data were analyzed by one-way ANOVA and multiple comparisons with Bonferroni’s correction. Letters “a” “b” “c” “d” mean significant differences between diet groups (P < 0.05). Only mean values of amino acids that showed statistical difference are presented, in alphabetic order.

**Figure 5:**
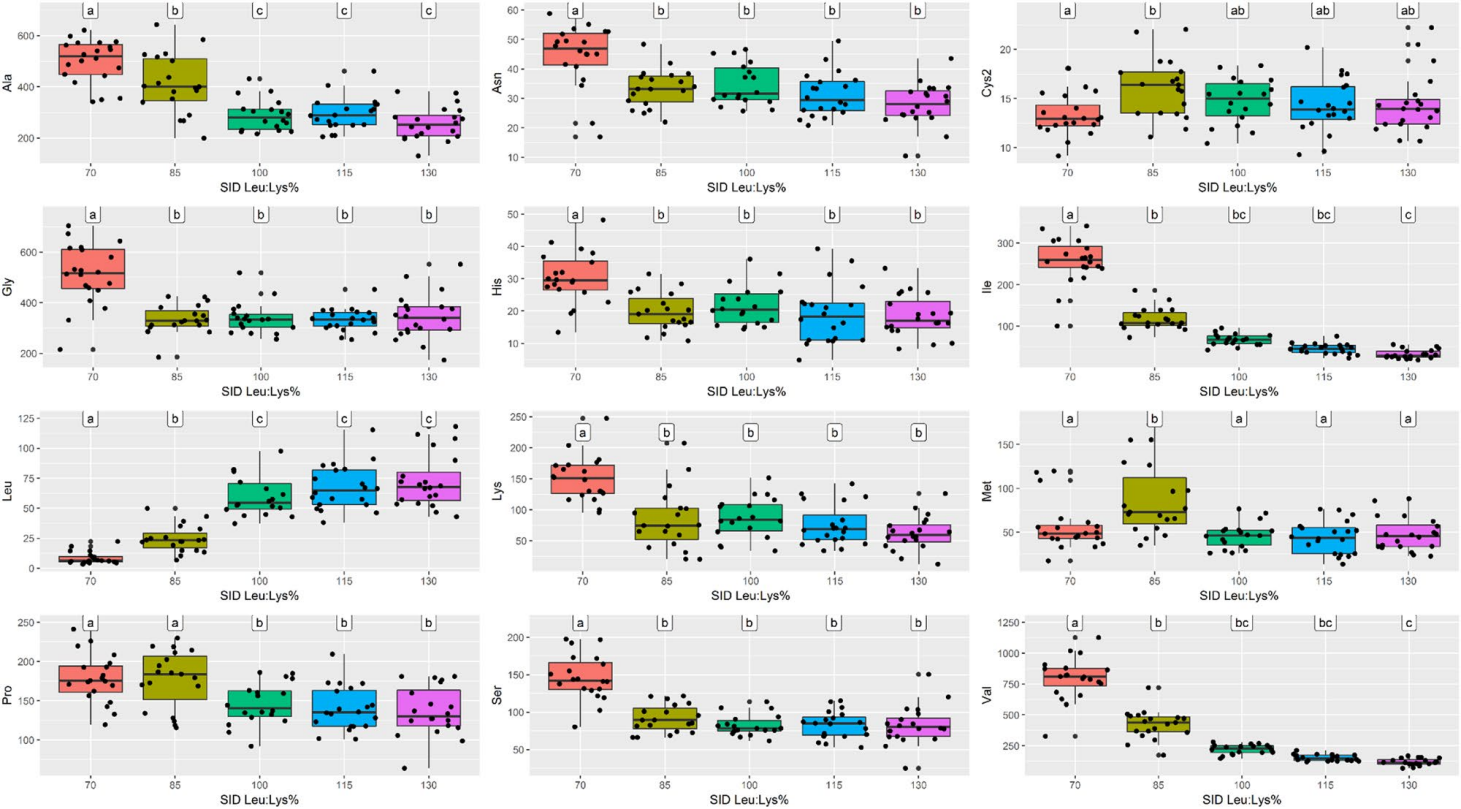
Amino acids concentration (μmol/l) in plasma at d28 associated to the different standardized ileal digestible leucine to lysine (SID Leu:Lys) dietary contents. Values represents the median, first and third quartile and 95% confidence interval of median of plasma AA of pigs fed diets with 70%, 85%, 100%, 115% and 130% SID Leu:Lys. Data were analyzed by one-way ANOVA and multiple comparisons with Bonferroni’s correction. Letters “a” “b” “c” “d” mean significant differences between diet groups (P < 0.05). Only mean values of amino acids that showed statistical difference are presented, in alphabetic order.

No significant effect was observed for *AASS* genotype on AAs levels in tissues (data not shown). At d7 (baseline), AA plasma levels were not different between the dietary treatments (Supplementary Fig. 4), while differences were recorded at d21 (Fig. 4) and d28 (Fig. 5). At both d21 and d28, plasma Leu level resulted significantly lower at 70 and 85% SID Leu:Lys and significantly higher at 115 and 130% SID Leu:Lys (*P* < 0.05). On the contrary, at d21 and d28, levels of several AAs (including Lys, Ile, Val, etc.) were significantly higher at 70% and/or at 85% SID Leu:Lys than in the other diets (*P* *<* 0.05). Furthermore, at d21 raising dietary Leu highly decreased Ile plasma level from 100 to 130% SID Leu:Lys and Val plasma level at 130% SID Leu:Lys (*P* *<* 0.05). At d28, significant lower plasma levels of both Val and Ile were observed at 130% SID Leu:Lys, compared to the other Leu levels. (Fig. 4). Amino acids such as Thr, Ornithine (Orn), Arginine (Arg), Trp, Phenylalanine (Phe) and Glutamine (Gln) did not result in significant difference at both d21 and d28 (data not shown). In liver, muscle and urine, AAs concentrations followed the same trend, but slightly less evident (Supplementary Figs. 5, 6 and 7).

**Figure 6:**
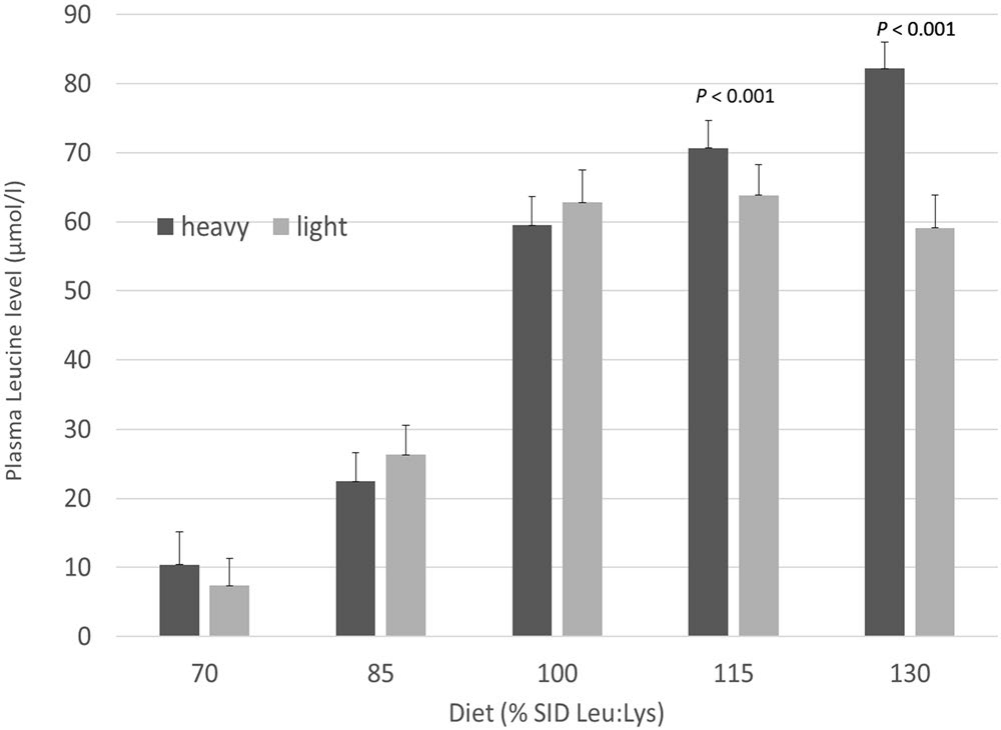
Plasma Leu level (μmol/l) at d28 for 2 body weight classes (light and heavy pigs) × 5 diets (70%, 85%, 100%, 115% and 130% SID Leu:Lys).

The plasma Leu level analysis in the 2 BW classes × 5 Diets design showed no differences at d7 and d21 (data not shown), whereas at d28 the interaction was significant between the plasma Leu concentration and the BW classes (Fig. 6). In particular, a significant difference (*P* < 0.001) in plasma Leu content between the two BW classes was observed at 115 and 130% SID Leu:Lys, with a lower content for the light compared to heavy pigs. No differences were seen for the other AAs between the two BW classes.

## Discussion

### Performance parameters

Previous dose-response studies have already reported effects of dietary Leu level on pig performances. On pigs weighing 10–20 kg, Gloaguen *et al*.^17^ observed that the increase from 70% to 86% SID Leu:Lys increased ADFI and ADG. Then, ADFI remained constant from 86% to 110%, while ADG increased up to 110% SID Leu:Lys. The increase of ADG from 102% to 110% SID Leu:Lys resulted mainly from an increase in feed efficiency whereas the response from 70% to 86% was mainly driven by the feed intake. Usually, feed intake response depends on the degree of AA deficiency. Animals are able to detect AA deficiencies and in general react by decreasing their feed intake^27^, although data on a compensation to a mild reduction of a single essential AA or, more often, of a group of essential AAs, with an increase of feed intake can be found in pigs^28^. Recently, a study on pigs of 10–28 kg BW reported a lower ADG in pigs fed 83% SID Leu:Lys compared to 94%, 104% and 115% SID Leu:Lys levels, where ADG was constant. No difference in ADFI and G:F between Leu levels was observed^18^.

In our study we found both similarities and differences for growth performance compared to the previous studies. ADG increased from 70% to 115% SID Leu:Lys which is in contrast with the previous observations^17,18^. This can be explained by the different BCAA levels, especially Val which was at 70 and 72% in the study by Wessels *et al*.^18^ and Gloaguen *et al*.^17^ respectively, and at 80% SID Val:Lys in our study, avoiding any limitation by this AA when Leu was increased. The decrease of ADG observed at 130% compared to 115% SID Leu:Lys agrees with what was reported by Wessels *et al*.^18^ over the 115% SID Leu:Lys level. We observed no reduction of ADFI when SID Leu:Lys increased, contrary to what was reported by García *et al*.^8^ where in pigs of 28–42 kg BW, an increase in Leu from 100% to 160% SID Leu:Lys decreased the ADFI in a dose-dependent manner. In our study, ADFI was lower at 70% SID Leu:Lys compared to others groups, while it did not change up to the 130% SID Leu:Lys. The F:G ratio constantly increased from 70% to 115% SID Leu:Lys, while at 130% we observed a decline of feed efficiency, accompanied by a decrease of ADG. It may reflect the effect of the dietary Leu excess on BCKDH activity, reducing the BCAAs availability^9,12^, and, in turn, probably leading to a Val and Ile deficiency. This could have counteracted the positive effect seen on muscle protein synthesis stimulation with less protein degradation observed with Leu supplementations^4–6^. When in excess, Leu may in fact lead to an imbalance in BCAAs and then to a reduction of feed intake^8–11^, with negative influence on growth performance^2,11^. This may indicate that for the pigs used in the present trial, the Leu requirement could have been slightly over the 100% SID Leu:Lys recommended by NRC^16^.

Actually, the LQ model used to calculate the Leu that maximizes ADG showed a value of 110.7% SID Leu:Lys, higher than the one calculated with the CLP model (100.5% SID Leu:Lys). The divergence between the two statistical models was already seen previously^18^, when the maximum response to Leu was estimated at 108% SID Leu:Lys using the LQ model and 104% using the CLP model. These authors noticed that LQ function better fitted their results, since the model considers the possible decline of the growth curve when nutrients exceed the maximizing dose. Despite the divergence in Leu maximizing dose obtained with the two models, considering the LQ model, the pig response in terms of ADG increased of +2.5%. For F:G, the LQ model fixed the response to Leu at 108.7% SID Leu:Lys which is in line with the results by Wessels *et al*.^18^ who estimated, using the LQ function, a value at 108.3% SID Leu:Lys to minimize the F:G ratio.

A Leu requirement identified in the range that maximized growth in this study (100.5 and 110.7% SID Leu:Lys) is higher than the value for Leu requirement defined and recommended by NRC^16^ for pigs of 7–11 and 11–25 kg of BW (100% SID Leu:Lys). It is also higher than 93% SID Leu:Lys, the value reported by Soumeh *et al*.^19^. On the contrary, in our range are the values reported by Gloaguen *et al*.^17^ and Wessels *et al*.^18^, who obtained a value of 102% and 108% SID Leu:Lys, respectively.

The *AASS* gene marker genotype of the piglets showed no effect on Leu:Lys requirements and growth performances. The plot of the prediction curves for ADG and F:G from the SID Leu:Lys dietary content (Supplementary Fig. 2) in general confirms that the response to dietary Leu level was not affected by the animal genotype. The investigated polymorphism marks a QTL region for basal level of plasma Lys in adult pigs, slaughtered at 155–160 kg live weights^25^. Therefore, it could be possible that the biological mechanisms controlling this AA level in the blood of slaughtering pigs do not interact with the dietary Leu:Lys ratio or are differently affected by the older age, that implies less protein deposition. Alternatively, those biological mechanisms may have been affected by the fasting practice imposed before slaughtering. Finally, it should be considered that the pigs were distributed in the groups balanced for the litters and for AASS genotype, but no other restriction was fixed in respects of their genetics. Thus, their variable genetic background could have had an overlapping effect on growth added to the effect of the selected gene. Further studies are needed to clarify these aspects.

### Body weight classes

Regarding the different responses in ADG to the different Leu levels seen in the two BW classes: for light pigs the LQ function estimated a maximum response at a Leu dose slightly lower than the CLP (110.5% and 115.4% SID Leu:Lys, respectively) while for heavy pigs, the LQ function estimated a Leu dose higher than that reported by the CLP model (110.2% and 94.9% SID Leu:Lys, respectively). The results for heavy pigs are in line with what reported by Wessels *et al*.^18^ and with what we found in this study considering all the pigs, where the LQ model estimated higher requirements than the CLP model. At the end, the LQ function estimated the same dose needed for the maximum response considering all the pigs or only the lights or only the heavy. On the contrary, the CLP model estimated a higher value for lights compared to heavy pigs with close to +7% more ADG for the light pigs when going from 94.9% to 115.4% SID Leu:Lys. The effect of BW classes on pig growth may be in some way interrelated with the dose-response to Leu. This might suggest that, at the same age, lighter pigs may require a higher SID Leu:Lys ratio to maximize their growth compared to heavier pigs. Among the references available on current web searching engines indexing scientific literature, no previous studies reported data on Leu requirements estimated in piglets with different weights at the same age. Nevertheless, it has been recently reported that dietary Leu supplementation increased body weight gain and skeletal muscle growth in intrauterine growth retardation (IUGR) piglets from 14 to 35 d of age, compared to IUGR piglets without Leu supplementation; conversely the same Leu supplementation did not increase growth in normal piglets^29^. These results would sustain our hypothesis that higher Leu dose in light pigs could support the growth. The use of a constant ideal AA profile has been previously questioned. In example, Mahan and Shield^30^ proposed that AA:Lys ratios may change with pig weight, age and genotype because of differences in protein deposition ratio between carcass and non-carcass components. Nevertheless, in a study by Ruiz-Ascacibar *et al*.^31^ on protein and AA deposition rates in pigs from 40 to 140 kg, the Leu:Lys ratio in body proteins was the same of that given by NRC^16^ and it did not change, showing that BW classes did not affect Leu:Lys ratio in pigs over 40 kg of BW. Therefore, further studies should be planned to better define and confirm the results we obtained that could open new tailored feeding practices in piglets based on their weight.

### Amino acid levels in plasma, liver, muscle and urine

The results of pig responses to different Leu dietary doses in terms of AAs concentration in tissues showed a strong effect of the treatment on AAs plasma level, in particular when Leu was highly deficient (70% SID Leu:Lys). Given its role as essential AA, the shortage of Leu led to a plasma increment of numerous AAs, both indispensable and not, such as Lys, Ile, Val, Asparagine (Asn), Glycine (Gly), Histidine (His), Proline (Pro), and Serine (Ser). These increased plasma values might presumably be due to the reduction of body protein synthesis, as consequence of insufficient supply of Leu amount to fulfill the potential for protein synthesis^6^. That might mean that these AAs, in these conditions, were not incorporated into proteins and remained free in the blood circulation, or that a possible body protein degradation happened to compensate a deficiency of one or more AAs, so increasing AA blood concentration. A similar response in terms of AA concentrations between 70% SID Leu:Lys and the other Leu dietary doses was seen in liver, muscle and urine, even if less evident than in plasma. The effect of Leu increment on BCAAs was also clear, with a strong reduction of Val and Ile concentrations in plasma. This pattern was already observed by García *et al*.^8^ and Wessels *et al*.^12^, due to a Leu excess that stimulates the BCKDH complex activity with a consequent increase in the BCAA catabolism^9^ and thus a decline of Val and Ile in blood. At 115% and 130% SID Leu:Lys, Leu plasma concentrations were significantly higher than at low Leu dietary values, as already reported by previous authors^8,9,12^ in pigs fed excess Leu in the diets, confirming that an increase in dietary Leu increases Leu plasma level.

Considering the plasma Leu level in the two BW classes at d28, significant differences between heavy and light pigs were observed, particularly when reaching 130% SID Leu:Lys in the two groups. On one hand, heavy pig plasma Leu concentration seems to keep saturating as long as the Leu:Lys ratio increases beyond the requirement whereas on the other hand, in light pigs there is a plateau of Leu in plasma once the requirement is exceeded. This plateau may lead to the hypothesis that, since Leu is one of the AAs most subjected to basal endogenous losses in pigs and these AA losses decrease as long as BW increases in growing pigs^32^, lighter pigs may have had greater endogenous losses of Leu than heavier pigs (which might also have increased Leu requirement), with no more Leu increase in plasma over the dose maximizing growth. On the other hand, the stabilization of plasma Leu level in light pigs may be due to the activity of BCKDH, whose action is highly stimulated when Leu is in excess leading to its own degradation^9,12^. Since we did not see significant differences in Val and Ile plasma concentrations in the two BW classes (data not shown), we hypothesized that decarboxylation by BCKDH could have more affected the Leu than the other BCAAs in this BW class.

## Conclusion

The SID Leu:Lys defined to increase weaned pig growth was higher than the value recommended by NRC^16^, but in line with other publications. The LQ function can better take into account the impact of Leu excess on weaned pig growth, but tests on larger range of Leu supplementation should be considered to confirm this observation. Our results on the different Leu requirements obtained in different BW classes are interesting for studies on a more focused and specific diet for animals differing for age and BW. Amino acid concentrations in tissues, especially in plasma, confirmed the importance of insuring a minimum Leu supply to support protein synthesis and its influence on the other BCAAs, when in excess.

## Methods

The procedures complied with the Italian law pertaining to experimental animals and were approved by the Ethic-Scientific Committee for Experiments on Animals of the University of Bologna and the Italian Ministry of Health by the approval number 479/2016-PR.

### Animals and study design

To investigate the effect of the dietary SID Leu:Lys level on growth performance and AA metabolism of weaned pigs, a 2 (Pig Genotype) × 5 (Diets) factorial design was performed on pigs differing for the two homozygous *AASS* gene region genotypes during three weeks of growth. The observed gene variation was an A/G Single Nucleotide Polymorphism, thus the two AASS homozygous genotypes were defined as “AA” and “GG”.

One-hundred piglets genotyped for an *AASS* gene marker were selected to have the two balanced genotypes equally distributed within litters, for a total of 50 pigs for each *AASS* homozygous genotype. Piglets were weaned averagely at 24 d of age (range between 22 d and 26 d) and stayed in the farm of origin for one week after weaning. Then, they were moved to the experimental facility of the Department of Agricultural and Food Science (DISTAL) - University of Bologna (d0) and divided in 10 groups (10 pigs/group), balanced for litter origin and BW (average BW of 8.2 ± 1.8 kg, mean ± SD).

From d0 to d2 the animals were kept in groups of two subjects per cage and then they were individually housed. Piglets were kept in cages with mesh floor, under controlled temperature and had free access to feed and water throughout the trial. We monitored diarrhea occurrence during the trial because it is a common health indicator in the weaning phase.

For the first week (d0 – d6), all piglets received the same diet with SID Leu:Lys ratio of 100%; then the three weeks dose-response trial started (from d7 to d28) and pigs received the diet assigned for treatment among the five experimental diets (average BW at d7: 9.3 ± 1.9 kg, mean ± SD).

### Diets

Five diets (Table 3) were formulated with different ratios of SID Leu:Lys, starting from the middle value of SID Leu:Lys at 100% as proposed by NRC^16^ for pigs of 7–11 kg and 11–25 kg of BW. The upper and lower values were established by increasing or decreasing SID Leu:Lys values by 15% and 30%, to obtain SID Leu:Lys levels of 70%, 85%, 115% and 130%. Diets were prepared starting from the lowest Leu content (SID Leu:Lys at 70%) and then adding progressively free L-Leu to reach the prefixed values. All the diets were formulated to contain 14.6% CP, 13.6 MJ ME/kg and a sub-limiting SID Lys level at 1.08% (about 80% of the recommendation of NRC^15^) in order to have co-limitation with Leu and to enable to show potential differences in Lys utilization. The other essential AAs were added to the calculated diets to meet the average recommendation suggested by NRC^16^ for pigs of 7–11 kg and 11–25 kg of BW; this means that the other essential AAs were not sub-limiting, contrary to what was for Lys. L-aspartate and L-glutamate were added to compensate the addition of L-Leu in the different diets and achieve equal CP concentrations between diets (Table 3). Diets did not include antimicrobials, zinc oxide or growth promoters. To design the diets, the nutrients values were estimated using EvaPig® software. Dietary CP and amino acid content of the intermediate diet (100% SID Leu:Lys) were analyzed as follows. Crude protein was calculated based on analyzed Nitrogen × 6.25. The nitrogen content was determined by DUMAS method following Standard NF EN ISO 16634–1. Total amino acids dosing methods were done according to European Commission regulation N° 152/2009 - NF EN ISO 13903: acid hydrolysis with HCl 6 N with reflux for 23 hours at 110 °C, followed by ion exchange chromatography on a specific amino acid analyser. Methionine and Cystine (Cys2) are subjected to a performic oxidation to avoid their damage during hydrolysis; Tyr and Phe are determined on the hydrolysis without oxidation. For total Trp, the method followed is MOD.0094 – Repealed standard AFNOR XP V18-114: alkaline hydrolysis with barium hydroxide for 16 hours in an autoclave at 120 °C. Separation by inverse phase high performance liquid chromatography and fluorometric detection.

**Table 3:** Experimental diets differing for standardized ileal digestible leucine to lysine ratio (SID Leu:Lys).

Diets	% SID Leu:Lys				
	70	85	100^1^	115	130
**Ingredient, %**					
Wheat, soft	57.00	57.00	57.00	57.00	57.00
Barley	16.06	16.06	16.06	16.06	16.06
Whey, sweet, dehydrated, skimmed	10.00	10.00	10.00	10.00	10.00
Soybean meal, 48% CP	5.00	5.00	5.00	5.00	5.00
Starch, maize	3.00	3.00	3.00	3.00	3.00
Calcium carbonate	1.00	1.00	1.00	1.00	1.00
Soybean oil	2.50	2.50	2.50	2.50	2.50
Monocalcium phosphate	1.25	1.25	1.25	1.25	1.25
L-Lysine HCl	0.77	0.77	0.77	0.77	0.77
Sodium chloride	0.45	0.45	0.45	0.45	0.45
L-Threonine	0.41	0.41	0.41	0.41	0.41
DL-Methionine	0.34	0.34	0.34	0.34	0.34
L-Valine	0.36	0.36	0.36	0.36	0.36
L-Phenylalanine	0.16	0.16	0.16	0.16	0.16
L-Histidine	0.12	0.12	0.12	0.12	0.12
L-Tryptophan	0.12	0.12	0.12	0.12	0.12
L-Isoleucine	0.20	0.20	0.20	0.20	0.20
L-Tyrosine	0.16	0.16	0.16	0.16	0.16
L-Leucine	**—**	**0.16**	**0.30**	**0.45**	**0.60**
L-Aspartate	**0.30**	**0.22**	**0.15**	**0.08**	—
L-Glutamate	**0.30**	**0.22**	**0.15**	**0.07**	—
Premix^2^	0.50	0.50	0.50	0.50	0.50

^1^Calculated values of 100% SID Leu:Lys diet (as fed): Crude protein, 14.55%; Crude fat, 3.45%; NDF, 10.43%; Calcium; 0.778%; Phosphorus, 0.633%; ME, 13.56 MJ/kg. Analyzed values (as fed): Dry matter, 90.68%; Leu, 1.29%; Lys, 1.22%; Arg, 0.76%; His, 0.41%; Ile, 0.78%; Met, 0.50%; Met + Cys, 0.74%; Phe, 0.79%; Phe + Tyr, 1.35%; Thr, 0.87%; Trp, 0.25%; Val, 0.99%; Ala, 0.57%; Asp, 1.30%; Glu,3.25%; Gly, 0.56%; Pro, 1.01%; Ser, 0.67%.

^2^The premixture supplied the following per kg complete diet: vitamin A, U.I. 12,000; vitamin D3, U.I. 2,000; vitamin E, 50 mg; vitamin K3 (MNB) 1.625; vitamin B1, 1.65 mg; vitamin B2, 5.375 mg; vitamin B6 (pyridoxine hydrochloride), 3.125 mg; vitamin B12, 0,05 mg; biotin, mg 0.2; choline chloride, 600 mg; niacine, 30 mg; folic acid, 2.50 mg; calcium D-panthotenate, 10 mg; copper (copper (II) sodium pentahydrate, 20 mg; iron (iron (II) sulphate monohydrate), 120 mg; iodine (anhydrous calcium iodide), 1 mg; manganese (manganese (II) oxide), 40 mg; zinc (zinc oxide); 100 mg; selenium (sodium selenite), 0.30 mg.

### Performance parameters and sampling

Cross breed pigs [(Large White × Landrace) × Duroc] were individually penned and weighted individually at the start of the trial (d0) and then weekly (d7, d14, d21, d28). Feed intake was daily recorded to calculate performance parameters.

Blood samplings were performed at d7, d21 and d28. The day of collection, the animals were fed with an amount of feed equal to 2% of their BW and feed was made available for twenty minutes. Then, eventual feed residue was removed and weighted. Four hours from the end of the meal, blood was collected from the external jugular vein using EDTA coated vacutainer tubes. Immediately after sampling, blood tubes were centrifuged at 3000 g at 4 °C for 10 min, then plasma was quickly dispensed into vials, snap frozen in liquid nitrogen and stored at −80 °C.

At the end of the trial (d28) all animals were sacrificed. Pigs were anesthetized with sodium thiopental (10 mg/kg BW) and sacrificed via an intracardiac injection of Tanax (0.5 ml/kg BW). Samples from liver, muscle (*biceps femoris*) and urine were collected. All samples were snap frozen in liquid nitrogen and stored at −80 °C.

### Genotyping of the piglets

Before weaning, genomic DNA of each pig was extracted from hair roots. Hair roots were incubated in Proteinase K solution (10 mg/mL of proteinase K in buffer [20 mM Tris HCl (pH 8.4), 50 mM KCl]) for 2 hours at 60 °C, then the proteinase was inactivated at 95 °C. Samples were stored at −20 °C. Samples were genotyped by PCR-RFLP for a DNA marker in the *AASS* gene region as reported by Fontanesi *et al*.^25^. Piglets with homozygous genotypes were selected for the trial.

### Biological samples deproteinization

Plasma, liver, muscle and urine samples were deproteinized. For plasma and urine, 200 μl of ice cold 5% Trichloroacetic acid (TCA) was added to 100 μL of the biofluid and mixed by vortex. Then, samples were centrifuged at 10000 rpm at 4 °C for 30 min and the supernatant was collected and stored at −80 °C.

For liver and muscle, 500 μL of ice cold 10% TCA was added to 0.1 g of tissue sample and homogenized. After 30 min of incubation on ice, samples were centrifuged at 14500 *g* at 4 °C for 30 min, the supernatant was filtered using a Millipore disk filter (Φ 20 μm) and the flow-through was collected and stored at −80 °C.

### Analyses of AA in tissues and urine

Plasma free AA were measured with UF-Amino Station system (Shimadzu Corporation, Kyoto Japan). Briefly, AA in deproteinized plasma were derivatized with 3-aminopyridyl-N-hydroxysuccinimidyl carbamate on line. The derivatized AA were separated by reverse phase chromatography and were detected by a LCMS-2020 single quadrupole mass spectrometer with ESI mode. Free AA in other tissues or urine samples were measured with L-8900 automated AA analyzer (Hitachi High-Technologies, Tokyo, Japan). Briefly, AA in deproteinized sample were separated by cation-exchange chromatography. Separated AA were detected spectrophotometrically after post column reaction with ninhydrin reagent.

### Statistical analyses

Performance parameters of the piglets were investigated using analysis of variance (ANOVA) of the GLM procedure of SAS with the 2 (Pig Genotype) × 5 (Diets) factorial design, corrected for the litter effect and including genotype and diets as fixed effects to evaluate the influence on growth performance.

Data were analyzed to estimate the SID Leu:Lys level needed to maximize ADG and F:G by regression analysis using linear (i) and quadratic (ii) models of the PROC REG, as well as LP (iii) and CLP (iv) models of the PROC NLIN and NLMIXED procedures of SAS^26^. The models are based on the following equations:

i $${Y}_{i}=aX+b$$

ii $${Y}_{i}=a{X}^{2}+bX+c$$

iii $$\begin{array}{rcl}{Y}_{i} & = & L+U\cdot (R\mbox{--}X)\,for\,X < R\\ {Y}_{i} & = & {Y}_{max}\,for\,X\ge R\end{array}$$

iv $$\begin{array}{rcl}{Y}_{i} & = & L+U\cdot (R\mbox{--}X)+V\cdot {(R\mbox{--}X)}^{2}\,for\,X < R\\ {Y}_{i} & = & {Y}_{max}\,for\,X\ge R\end{array}$$

The variable *Y*_*i*_ is the response of the *i*piglet, *Y*_*max*_ is the maximum response, *X* is the Leu dose, R is the breakpoint/plateau of the X values, a, b, c, L, U and V are the regression coefficients. The AASS genotype was also added as a fixed factor to (ii) model to test the possible variation of the response to Leu.

Analysis was performed on the full set of data and on the two separate genotypes. Furthermore, SID Leu:Lys doses to maximize ADG and F:G were estimated in two classes of BW. The two BW classes were defined based on values after the first week and composed of all pigs under and over the mean (light pigs initial BW: 7.9 ± 1.46 kg, mean ± SD; heavy pigs initial BW: 10.5 ± 1.12 kg, mean ± SD). Data are presented as least squares means and SEM.

Amino acid concentrations in blood (plasma), muscle, liver and urine were analyzed by one-factor ANOVA, considering Leu level as one factor, and then by multiple comparisons with significant thresholds adjusted using the Bonferroni’s correction. Amino acid levels in plasma were furthermore analyzed by GLM procedure of SAS with the 2 BW classes × 5 Diets design.

### Ethics approval

The procedures complied with the Italian law pertaining to experimental animals and were approved by the Ethic-Scientific Committee for Experiments on Animals of the University of Bologna and the Italian Ministry of Health by the approval number 479/2016-PR.

## Supplementary information

Supplementary Material
