# Supplementary material for: Dose-response of different dietary leucine levels on growth performance and amino acid metabolism in piglets differing for aminoadipate-semialdehyde synthase genotypes
Source: Sci Rep. 2019 Dec 6;9:18496. doi: 10.1038/s41598-019-55006-z (PMC6898585; doi:10.1038/s41598-019-55006-z)
Supplement: Supplementary file 1 — Supplementary Material [file 41598_2019_55006_MOESM1_ESM.docx]

Dose-response of different dietary leucine levels on growth performance and amino acid metabolism in piglets differing for aminoadipate-semialdehyde synthase genotypes.

Micol Bertocchi^1^, Paolo Bosi^2^, Diana Luise^2^, Vincenzo Motta^2^, Chiara Salvarani^2^, Anisa Ribani^2^, Samuele Bovo^2^, Aude Simongiovanni^3^, Keiko Matsunaga^4^_,_ Tetsuya Takimoto^4^, Makoto Bannai^4^, Etienne Corrent^3^, Luca Fontanesi^2^, Tristan Chalvon-Demersay^3^, Paolo Trevisi^2^*

^1^*Department of Agricultural, Environmental and Food Sciences, University of Molise, Campobasso, Italy*

^2^*Department of Agricultural and Food Sciences, University of Bologna, 40127 Bologna, Italy*

^3^*Ajinomoto Eurolysine S.A.S., 75017 Paris, France*

^4^*Ajinomoto Co., Inc., Tokyo 104-8315, Japan*


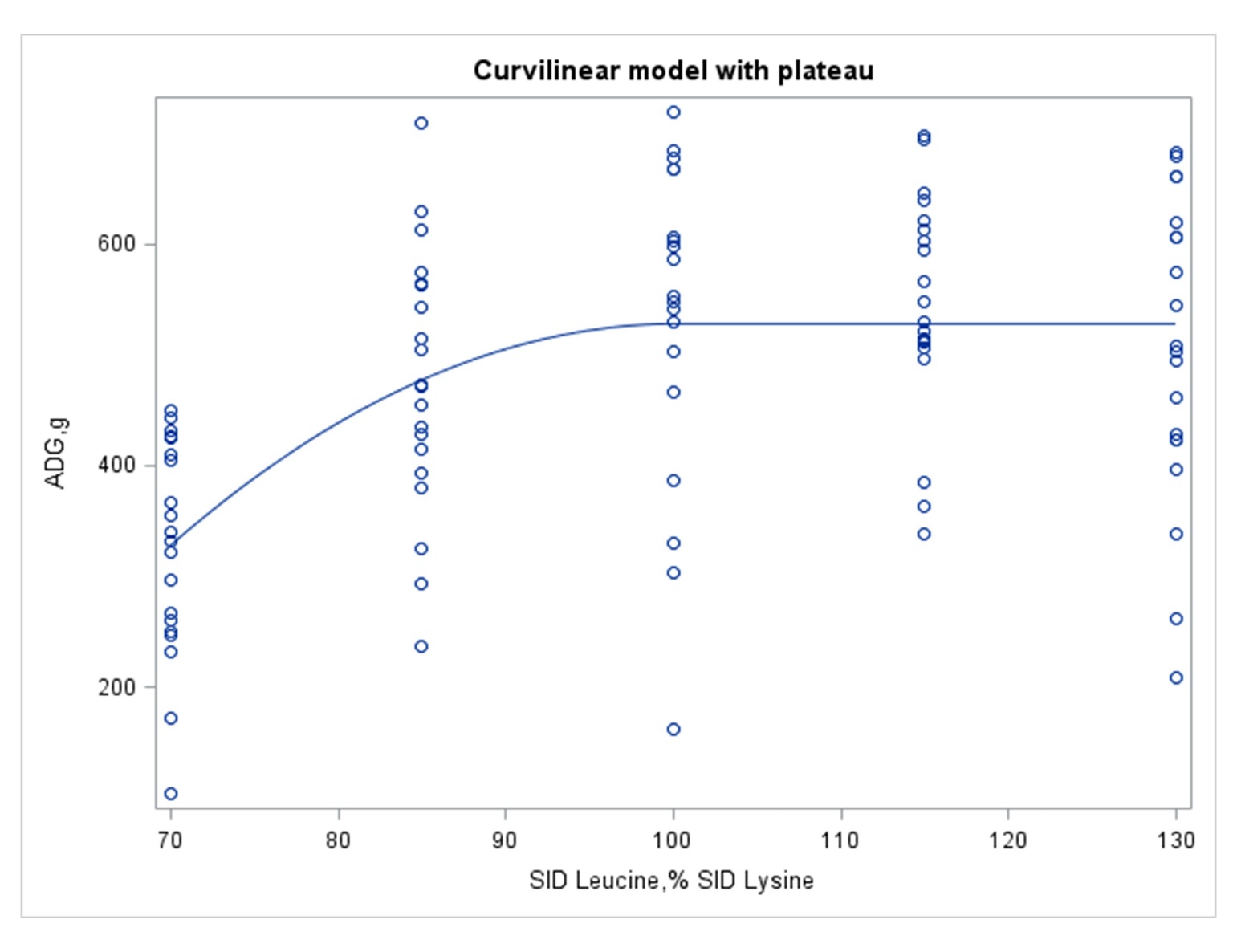


**Supplementary figure 1.** **Estimation of leucine requirement for weaned piglets. Prediction of the average daily gain (ADG) according to the standardized ileal digestible (SID) leucine to lysine dietary content with a curvilinear-plateau model.** Observed values (**o**) from d7 to end (d28). Equation of prediction before inflection (parameters ± S.E.): Y= -1653 (± 905) + 43.61 (± 21.44)*X – 0.2170 (± 0.1244)*X^2^. Predicted inflection point at 538 g ADG with 100.5% SID leucine to lysine. Adjusted R^2^ = 0.327.


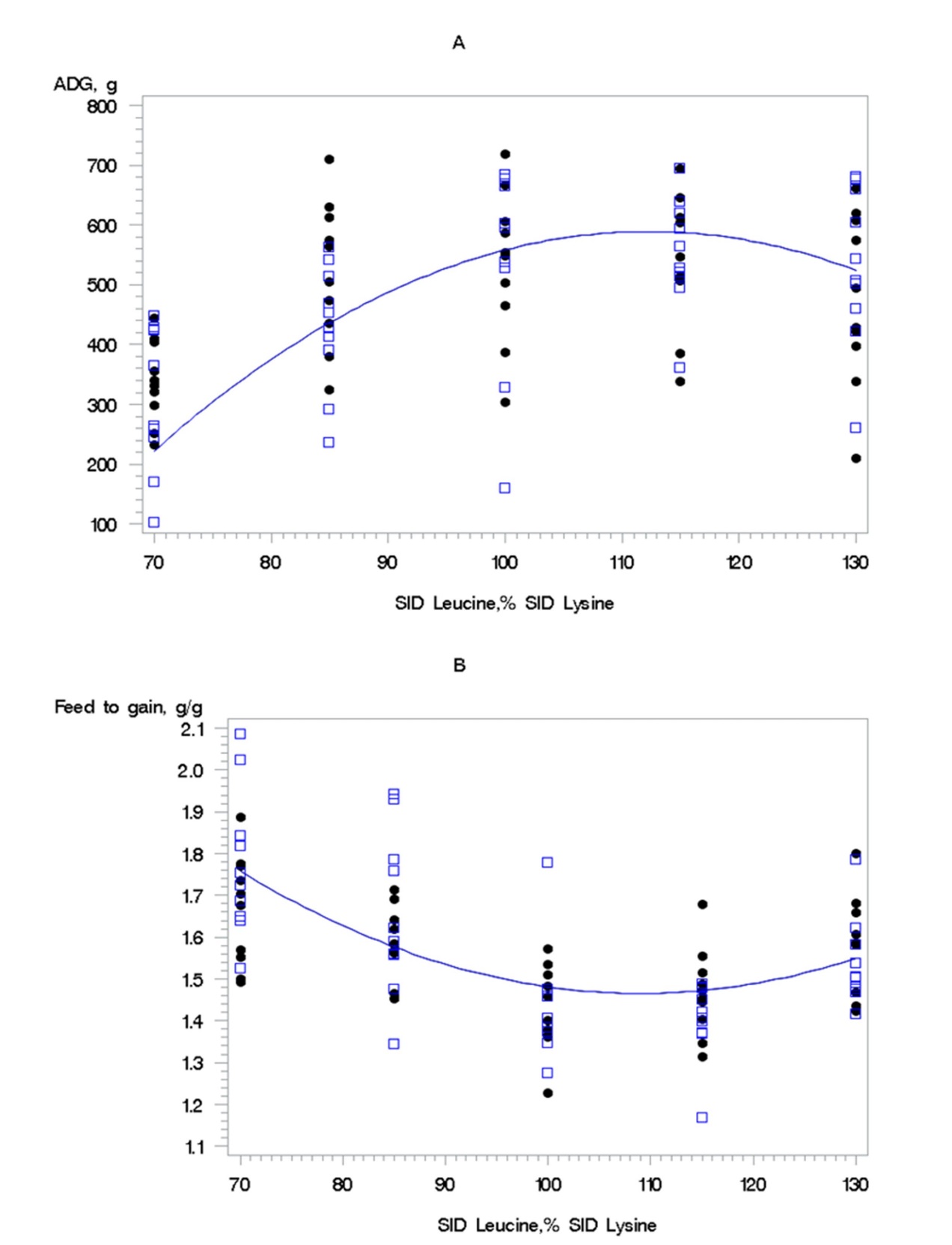


**Supplementary Figure 2. Plot of observed values for the two AASS genotypes against the quadratic prediction for average daily gain (ADG, A) and feed to gain (B) according to the standardized ileal digestible (SID) leucine to lysine dietary content.** The prediction lines for each AASS genotype are overlaying. (•) AASS=”AA”; (**□**) AASS=”GG”.


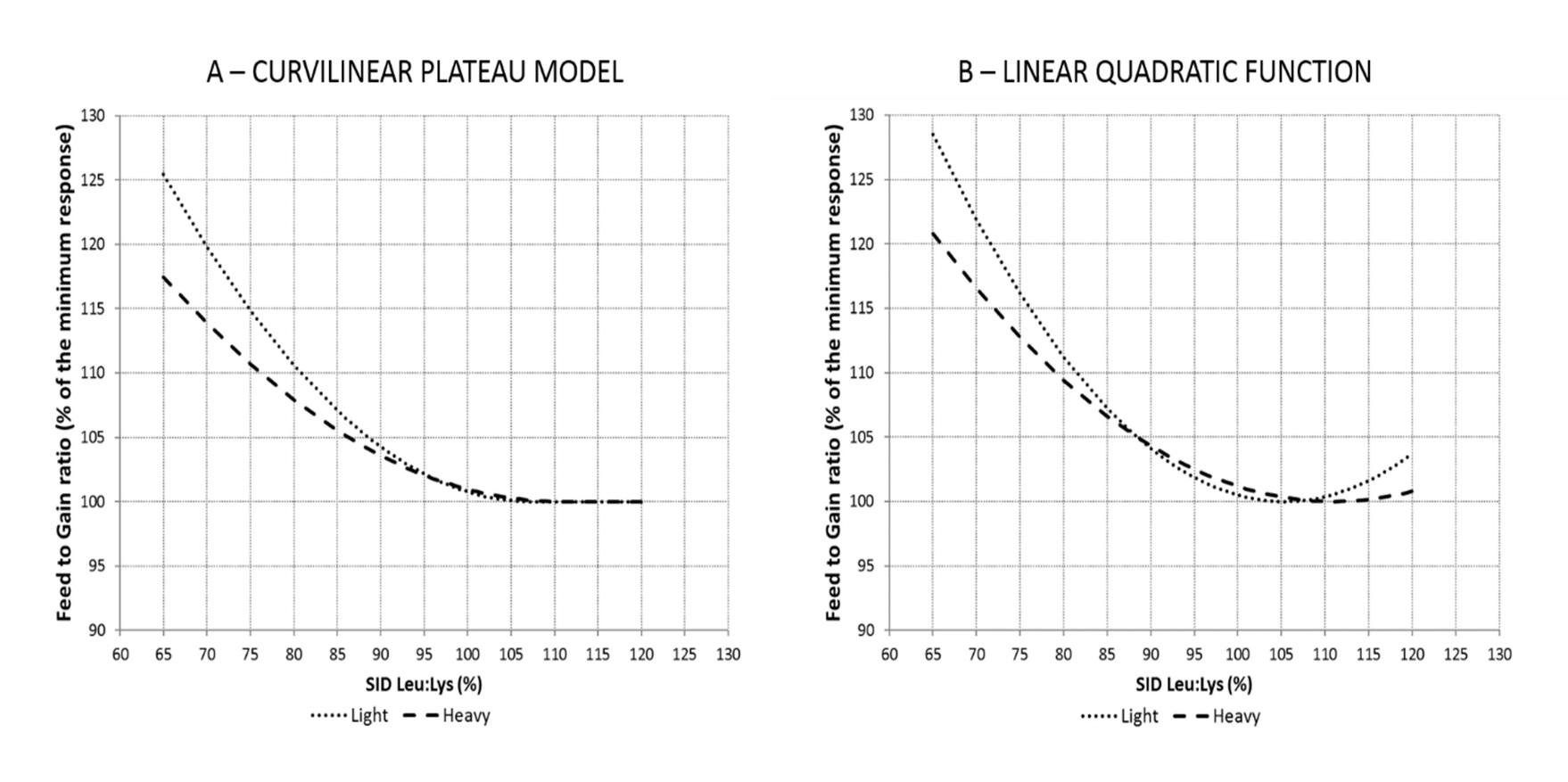


**Supplementary Figure 3. Estimation of leucine requirement for weaned piglets. Percentage of response for feed to gain ratio according to the standardized ileal digestible (SID) leucine to lysine dietary content** **predicted by curvilinear-plateau model (A) and linear quadratic function (B) in 2 different body weight classes (light and heavy pigs) from d7 to end (d28).** (A) Equation of prediction in light pigs (• • •) before inflection (parameters ± S.E.): Y = 3.904 (± 1.185) - 0.0451 (± 0.0273)***X + 0.00021 (± 0.00015)***X^2^. Adjusted R^2^ = 0.369; Root mean square error (RMSE) = 0.116; Estimated inflection point = 1.49 of feed to gain with 106.9% SID leucine to lysine. Equation of prediction in heavy pigs (− − −) before inflection (parameters ± S.E.): Y = 3.153 (± 0.7903) - 0.0288 (± 0.0175)***X + 0.00013 (± 0.00009)***X^2^. Adjusted R^2^ = 0.305; Root mean square error (RMSE) = 0.104; Estimated inflection point = 1.53 of feed to gain with 112.4% SID leucine to lysine. (B) Equation of prediction in light pigs (• • •) (parameters ± S.E.): Y = 4.375 (± 0.494) - 0.0548 (± 0.0103)*X + 0.00026 (± 0.00005)*X^2^. Adjusted R^2^ = 0.461; Root mean square error (RMSE) = 0.099; Estimated minimum point at 1.44 feed to gain with 107.0% SID leucine to lysine. Equation of prediction in heavy pigs (− − −) (parameters ± S.E.): Y = 3.373 (± 0.453) - 0.0333 (± 0.0093)*X + 0.00015 (± 0.00005)*X^2^. Adjusted R^2^ = 0.332; Root mean square error (RMSE) = 0.100; Estimated minimum point at 1.53 feed to gain with 110.3% SID leucine to lysine.


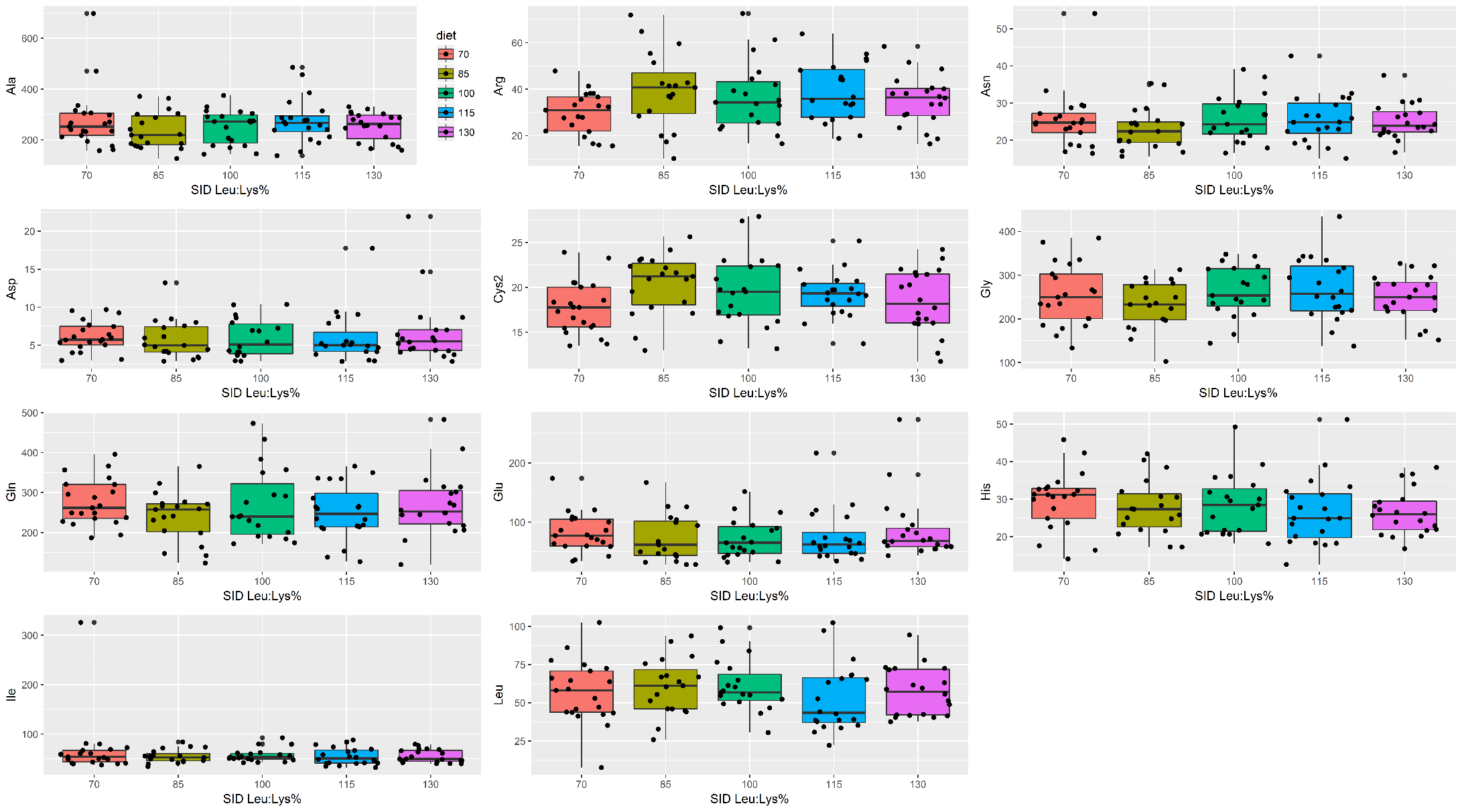


Supplementary Figure 4 (A)


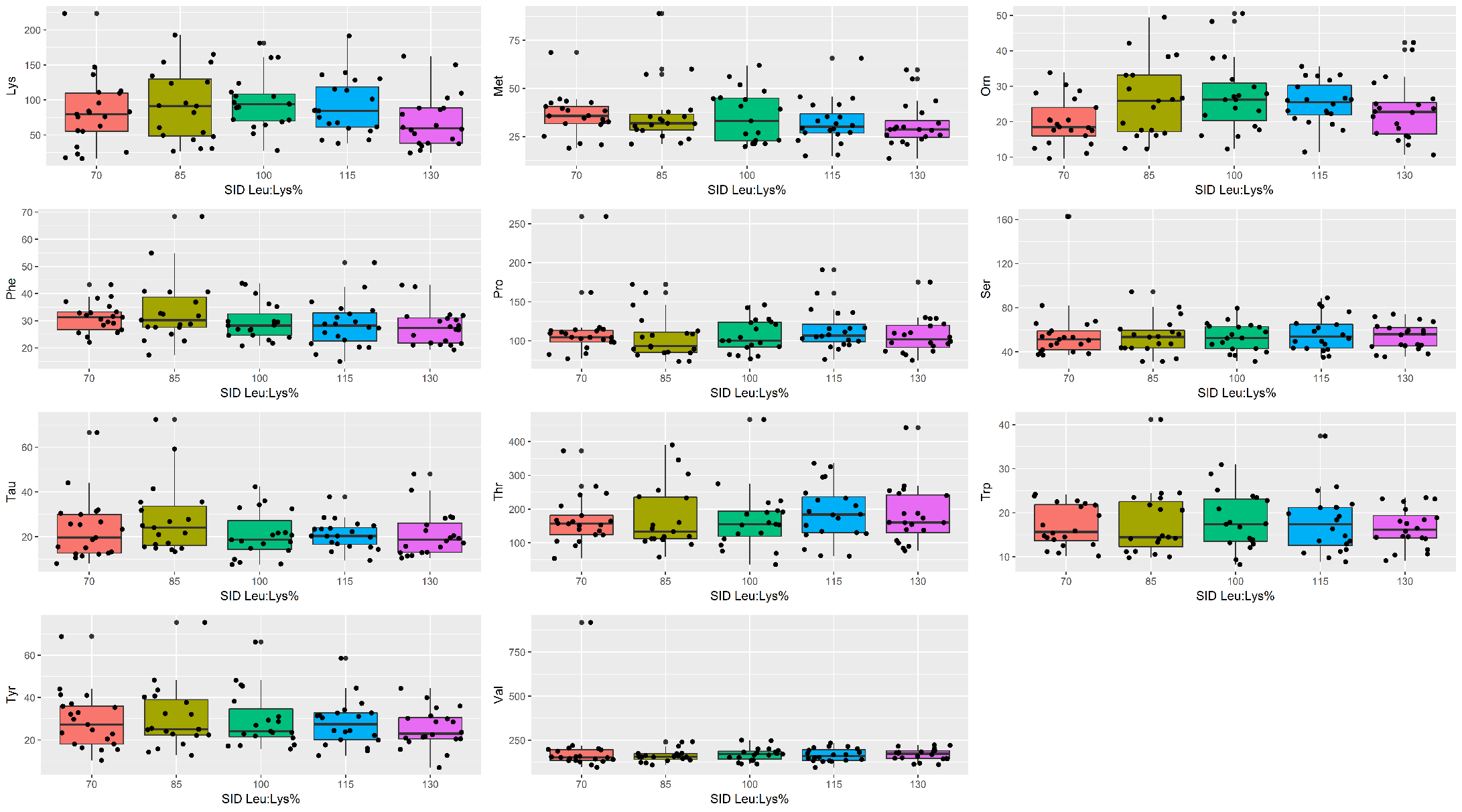


Supplementary Figure 4 (B).

**Supplementary Figure 4 (A) and (B). Amino acids concentration (μmol/l) in plasma at day 7 (baseline) associated to the different leucine dietary contents (% on SID lysine).** Values represents the median, first and third quartile and 95% confidence interval of median of pigs fed diets with 70%, 85%, 100%, 115% and 130% SID Leu:Lys. Data were analyzed by one-way ANOVA and multiple comparisons with adjustment Bonferroni. Letters “a” “b” “c” “d” mean significant differences between diet groups (P < 0.05). Only the statistically significant AA were reported together with those remaining that were considered more interesting.


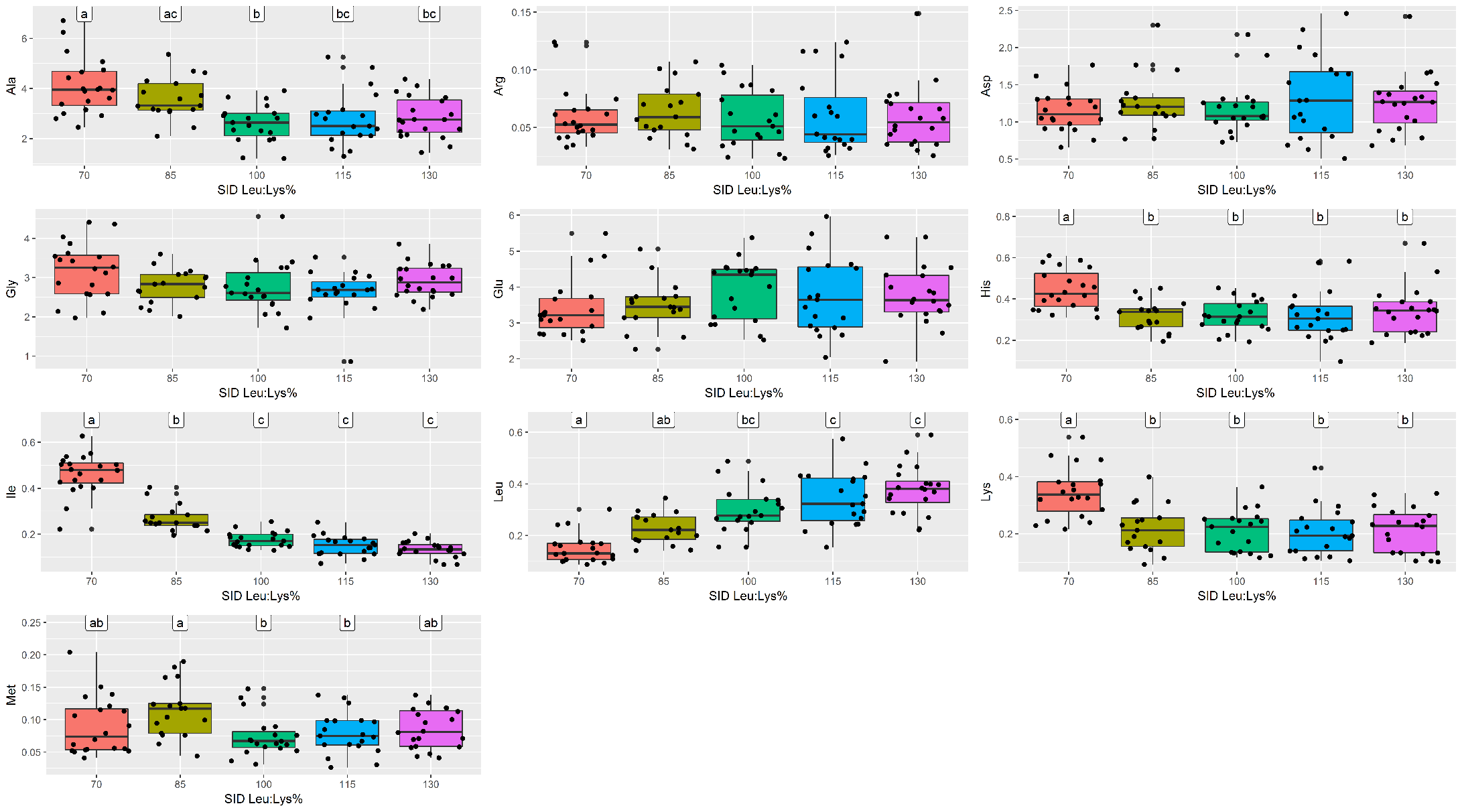


Supplementary Figure 5 (A)


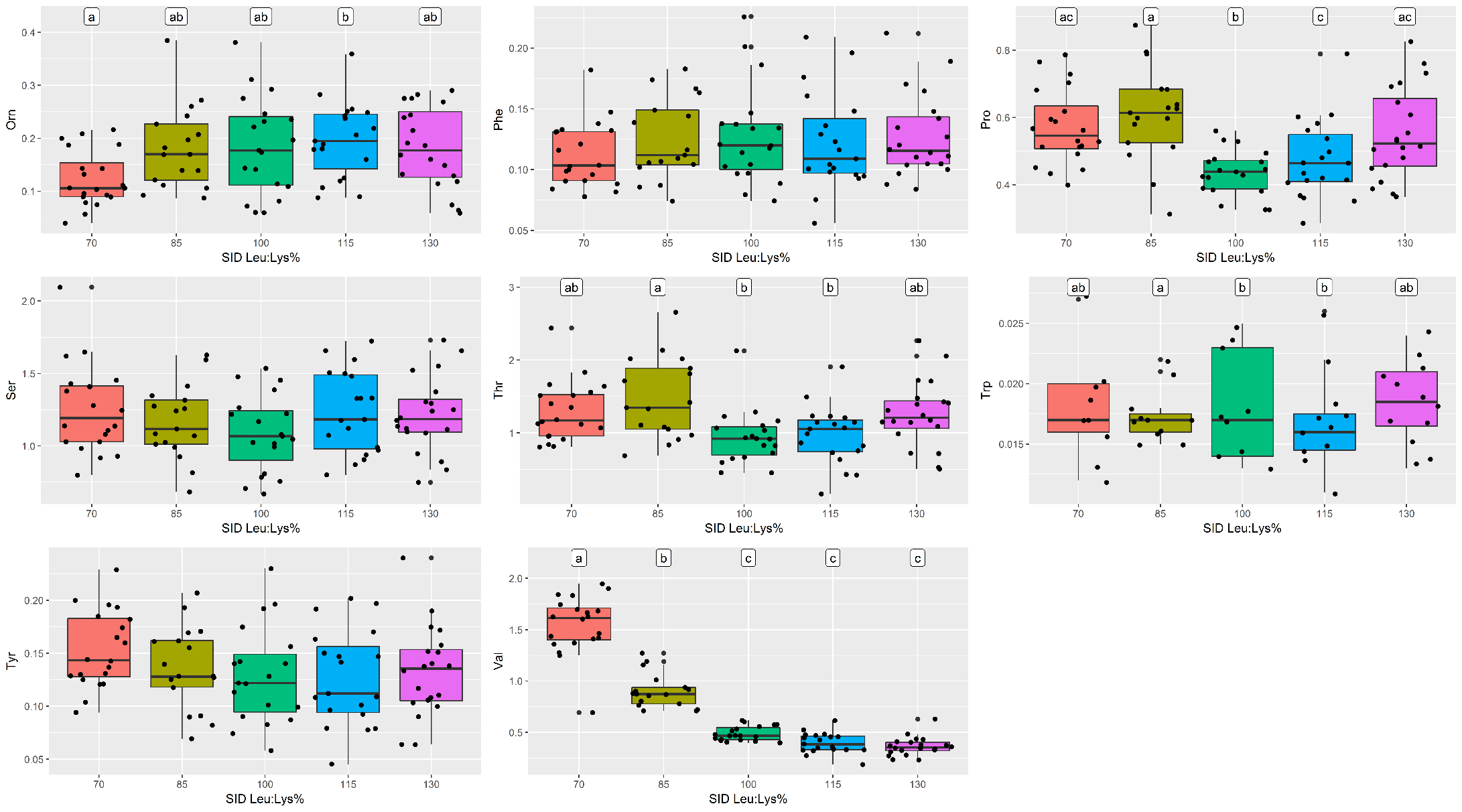


Supplementary Figure 5 (B).

**Supplementary Figure 5 (A) and (B). Amino acids concentration (μmol/g) in liver (d 28) associated to the different leucine dietary contents (% on SID Lysine).** Values represents the median, first and third quartile and 95% confidence interval of median of pigs fed diets with 70%, 85%, 100%, 115% and 130% SID Leu:Lys. Data were analyzed by one-way ANOVA and multiple comparisons with adjustment Bonferroni. Letters “a” “b” “c” “d” mean significant differences between diet groups (P < 0.05). Only the statistically significant AA were reported together with those remaining that were considered more interesting.


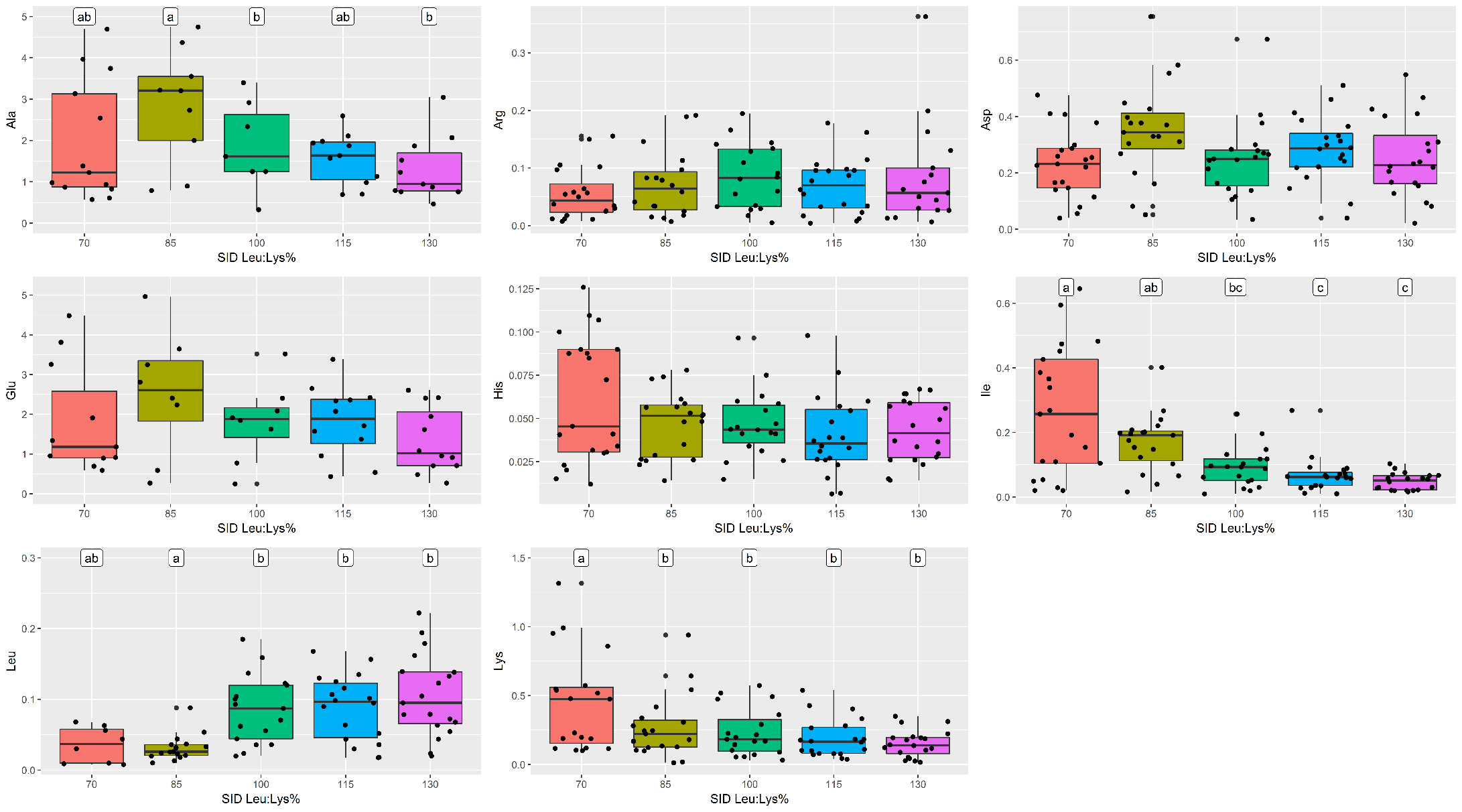


Supplementary Figure 6 (A)


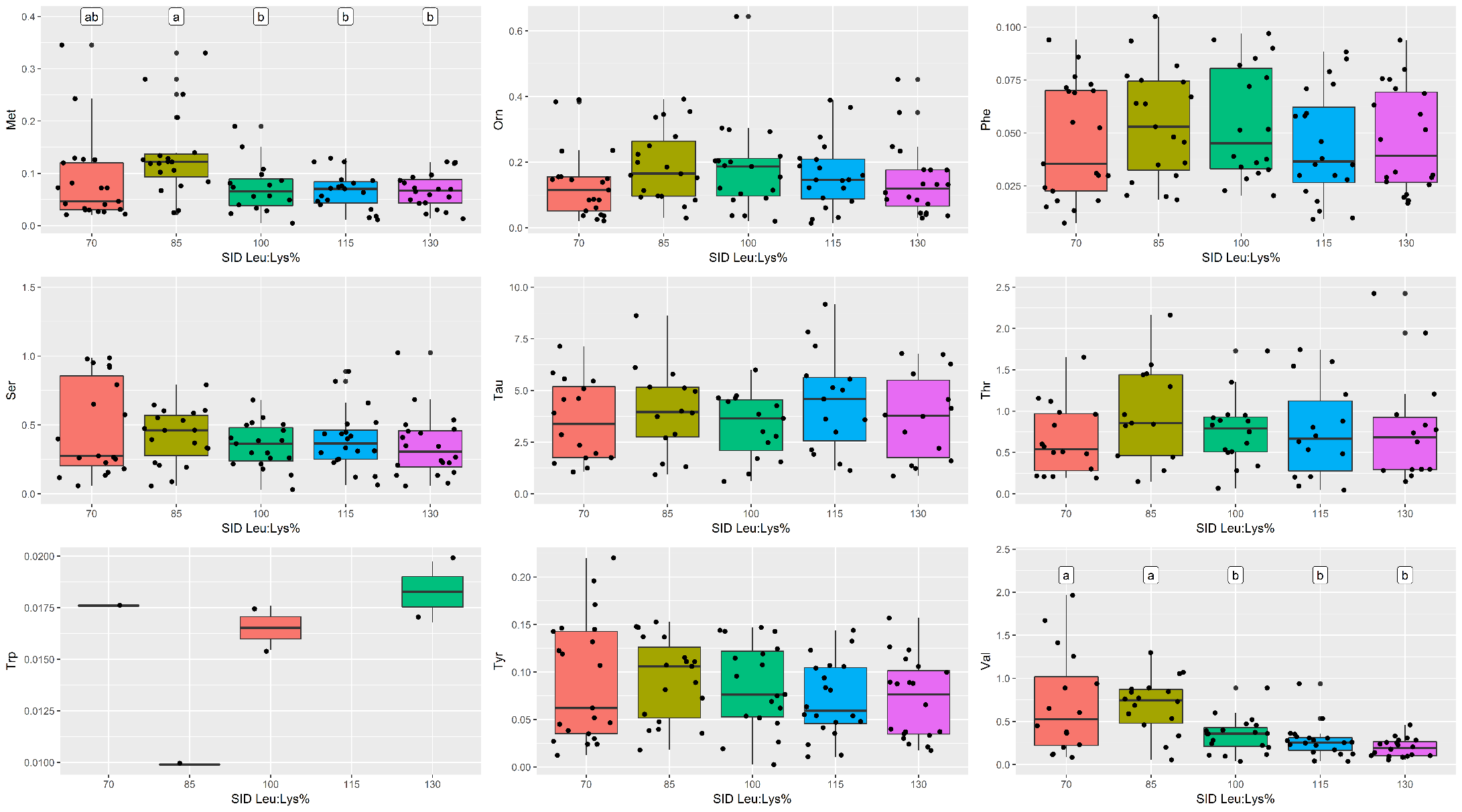
Supplementary Figure 6 (B).

**Supplementary Figure 6 (A) and (B). Amino acids concentration (μmol/g) in muscle (d28) associated to the different leucine dietary contents (% on SID lysine)**. Values represents the median, first and third quartile and 95% confidence interval of median of pigs fed diets with 70%, 85%, 100%, 115% and 130% SID Leu:Lys. Data were analyzed by one-way ANOVA and multiple comparisons with adjustment Bonferroni. Letters “a” “b” “c” “d” mean significant differences between diet groups (P < 0.05). Only the statistically significant AA were reported together with those remaining that were considered more interesting.


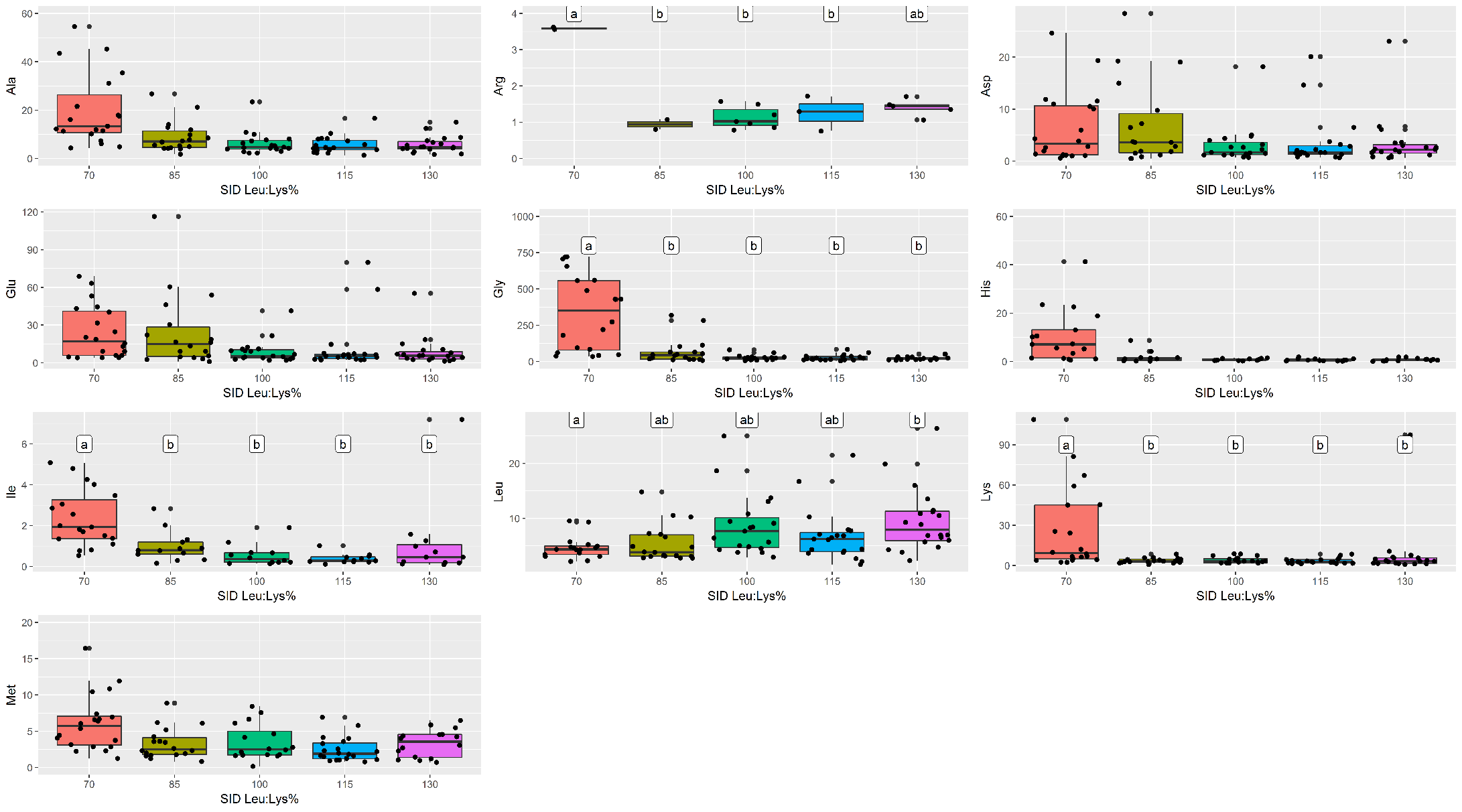


Supplementary Figure 7 (A)


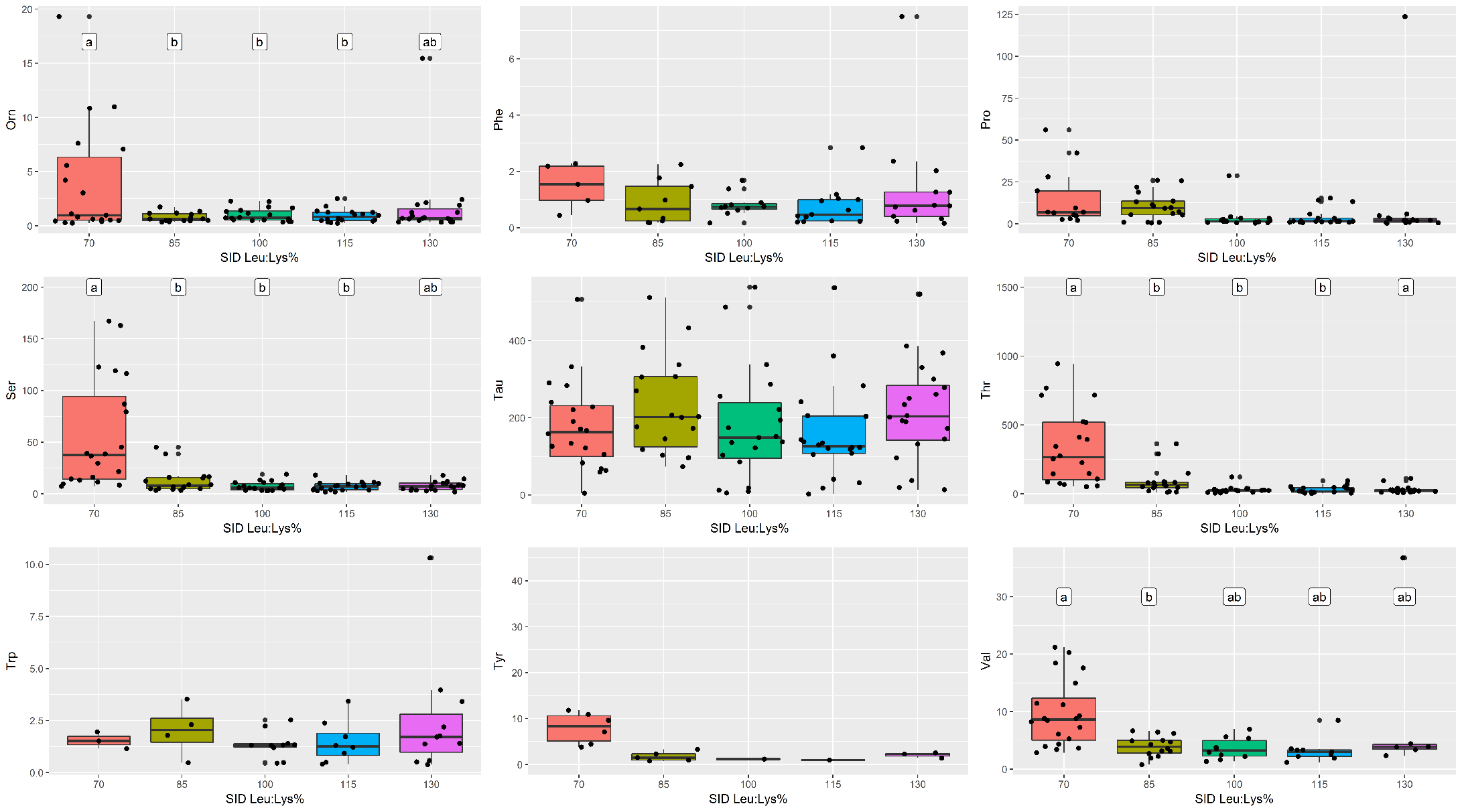
Supplementary Figure 7 (B).

**Supplementary Figure 7 (A) and (B). Amino acids concentration (μmol/dl) in urine (d28) associated to the different leucine dietary content (% on SID lysine).** Values represents the median, first and third quartile and 95% confidence interval of median of pigs fed diets with 70, 85, 100, 115, 130% SID Leu:Lys. Data were analyzed by one-way ANOVA and multiple comparisons with adjustment Bonferroni. Letters “a” “b” “c” “d” mean significant differences between diet groups (P < 0.05). Only the statistically significant AA were reported together with those remaining that were considered more interesting.
